# Combination of selective androgen and estrogen receptor modulators in orchiectomized rats

**DOI:** 10.1007/s40618-022-01794-7

**Published:** 2022-04-16

**Authors:** P. J. Roch, V. Wolgast, M.-M. Gebhardt, K. O. Böker, D. B. Hoffmann, D. Saul, A. F. Schilling, S. Sehmisch, M. Komrakova

**Affiliations:** 1 Department of Trauma Surgery, Orthopaedics and Plastic Surgery, University of Göttingen, Robert-Koch-Str. 40, 37075 Göttingen, Germany; 2 Kogod Center On Aging and Division of Endocrinology, Mayo Clinic, Rochester, MN 55905 USA; 3 Department of Trauma Surgery, Hannover Medical School, University of Hannover, Carl-Neuberg-Str. 1, 30625 Hannover, Germany

**Keywords:** Orchiectomized rat model, Selective androgen receptor modulators (SARMs), Selective estrogen receptor modulators (SERMs), Muscle, Bone

## Abstract

**Purpose:**

Selective androgen and estrogen receptor modulators, ostarine (OST) and raloxifen (RAL), reportedly improve muscle tissue and offer therapeutic approaches to muscle maintenance in the elderly. The present study evaluated the effects of OST and RAL and their combination on musculoskeletal tissue in orchiectomized rats.

**Methods:**

Eight-month-old Sprague Dawley rats were analyzed. Experiment I: (1) Untreated non-orchiectomized rats (Non-ORX), (2) untreated orchiectomized rats (ORX), (3) ORX rats treated with OST during weeks 0–18 (OST-P), (4) ORX rats treated with OST during weeks 12–18 (OST-T). Experiment II: 1) Non-ORX, (2) ORX, 3) OST-P, (4) ORX rats treated with RAL, during weeks 0–18 (RAL-P), 5) ORX rats treated with OST + RAL, weeks 0–18 (OST + RAL-P). The average daily doses of OST and RAL were 0.4 and 7 mg/kg body weight (BW). Weight, fiber size, and capillarization of muscles, gene expression, serum markers and the lumbar vertebral body were analyzed.

**Results:**

OST-P exerted favorable effects on muscle weight, expression of myostatin and insulin growth factor-1, but increased prostate weight. OST-T partially improved muscle parameters, showing less effect on the prostate. RAL-P did not show anabolic effects on muscles but improved body constitution by reducing abdominal area, food intake, and BW. OST + RAL-P had an anabolic impact on muscle, reduced androgenic effect on the prostate, and normalized food intake. OST and RAL improved osteoporotic bone.

**Conclusions:**

The OST + RAL treatment appeared to be a promising option in the treatment of androgen-deficient conditions and showed fewer side effects than the respective single treatments.

## Introduction

In the world’s aging population, a decline in muscle mass and function is of increasing importance. Due to the subsequent physical limitations of the elderly, the risk of falling is heightened. Hormonal changes in the aging organism, especially the decline of sex hormones such as testosterone and estrogen, severely impacts the complex interaction between the central nervous system, muscle, and bone [1–3].

Direct hormone replacement therapy with either testosterone or estrogen is an effective treatment for hormone deficiency in men and women [4–6]. However, both replacement therapies have been shown to be associated with severe side effects [5, 7]. Consequently, due to higher tissue selectivity, selective androgen and estrogen receptor modulators (SARM and SERM, respectively) offer new treatment options for the musculoskeletal system [2]. SARMs resist aromatization of 5-α-reduction compared to testosterone and are considered to have a better bioavailability and pharmacokinetic profile than testosterone [8]. Nevertheless, there has been no indication for their use so far [9]. SERMs present an established therapy for postmenopausal symptoms that have fewer side effects compared to estrogen [2]. In regard to musculature, studies reported improved muscle function and structure after SERM administration in male mice with muscular dystrophy [10, 11].

Ostarine (OST), also labeled as enobosarm, S-22, MK-2866, or GTx-024, is a SARM that has been clinically shown to have beneficial effects on body mass, muscles, and physical function [12–14]. In a postmenopausal rat model, OST increased vascularization and the activity of citrate synthase in skeletal muscles [15]. Raloxifene (RAL) is a SERM that treatment with which is associated with increased serum levels of testosterone and estrogen in elderly men [16, 17]. Furthermore, RAL administration was demonstrated to significantly increase fat-free body mass in postmenopausal women [18]. In female and male mice with muscular dystrophy, RAL ameliorated skeletal muscle function and structure [11].

Age-related declines in gonadal hormone levels and androgen deprivation in patients with prostate cancer have been shown to be associated to the development of sarcopenia [19, 20]. To our knowledge, no studies have yet reported the in vivo effects of combined OST and RAL treatment on muscle structure and metabolism under androgen deficient conditions as a model for sarcopenia. In an ovariectomized rat model for osteoporosis, a combination of SARM and SERM was shown to exert beneficial effects on bone [21]. In the present study, two independent experiments were conducted to examine the effects of OST and RAL and their combined treatment on skeletal muscle in orchiectomized rat model for androgen deficient conditions.

## Materials and methods

### General procedures

**Fig. 1:**
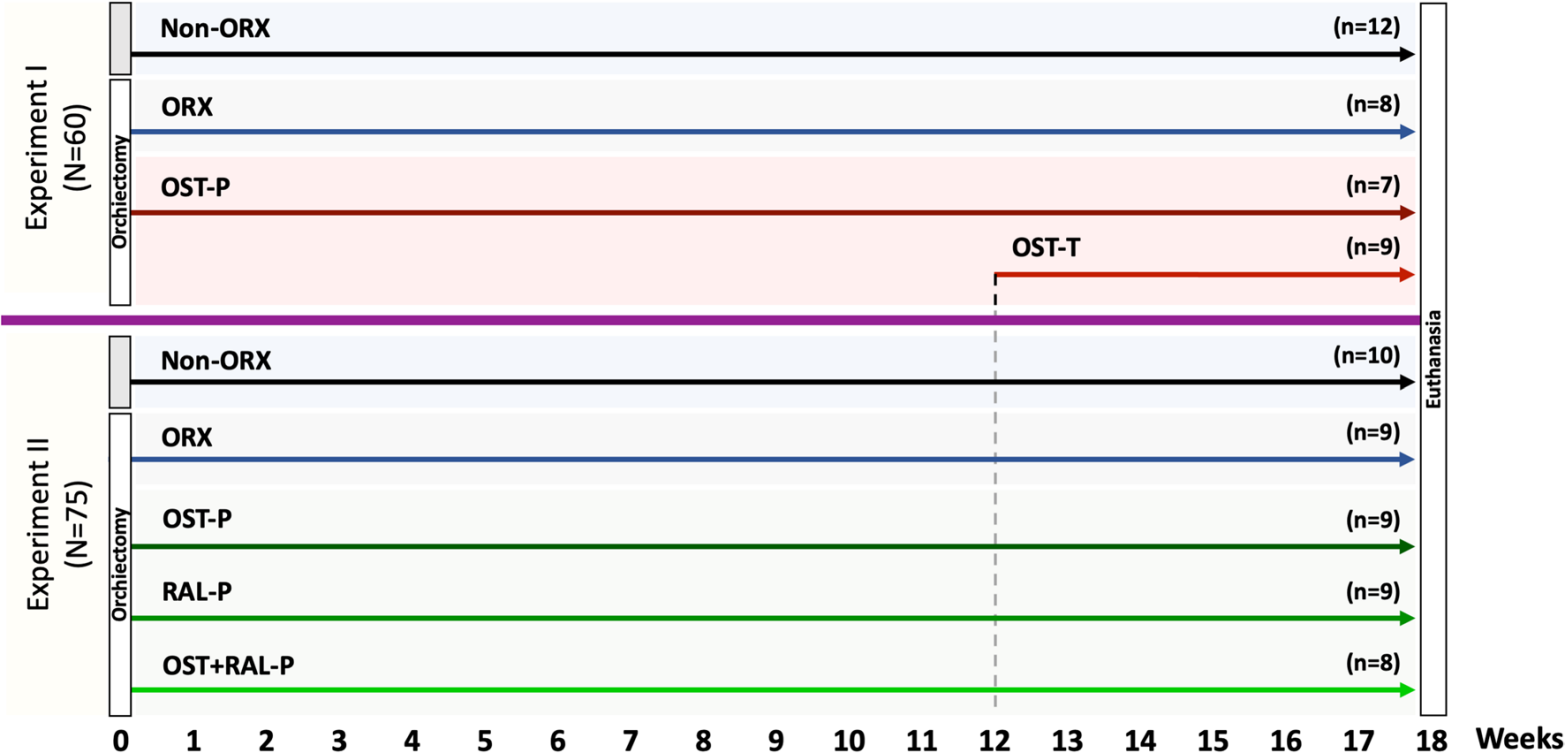
Schematic flowchart of the two experiments. Experiment I: Prophylactical (P) and therapeutical (T) OST administration; Experiment II: Prophylactical OST, RAL, and combined OST + RAL administration. Eight-month-old male rats were either orchiectomized (ORX) or left intact (Non-ORX). In Experiment I, one of the two ORX groups was treated with OST for 18 weeks after ORX (OST-P), and the other ORX group was treated with OST from weeks 12 to 18 after ORX (OST-T). In Experiment II, ORX rats were treated with OST, RAL, or OST + RAL for 18 weeks after ORX. At the beginning, each group in both experiments contained 15 rats (N). At the end of the experiment, (n) the number of rats was analyzed

The animal study protocol was approved by the local regional government (14/1396, Oldenburg, Germany) prior to the study. In total, 135 eight-month-old Sprague Dawley rats (Fa. Janvier Labs, Saint-Berthevin, France) were used in two experiments. At the beginning of both experiments, the rats were bilaterally orchiectomized (ORX) or left intact to serve as controls (Non-ORX). Thereafter, the rats were treated according to the experimental design (Fig. 1).

In Experiment I, the effect of OST applied immediately after ORX (prophylactically, OST-P) on muscle structure and metabolism was studied and compared with therapeutic treatment (OST-T) initiated 12 weeks after ORX. Four rat groups were randomized (Fig. 1)as follows: Group 1, Non-ORX (*n* = 15); Group 2, ORX (*n* = 15); Group 3, OST-P (*n* = 15), and Group 4, OST-T (*n* = 15). In the OST-P group, ORX rats were treated with OST immediately after ORX for up to 18 weeks, whereas in the OST-T group, they were treated 12 weeks after ORX for up to six weeks. The average dosage of OST was 0.4 mg/kg body weight (BW).

In Experiment II, the effects of OST, RAL, and a combination of both on muscle were studied. Rats were divided into the following five groups (Fig. 1): Group 1, Non-ORX (*n* = 15); Group 2, ORX (*n* = 15), and Groups 3 to 5, ORX rats treated with OST, RAL, or a combination of both (RAL + OST), with a dosage of 0.4 mg/kg BW for OST and 7 mg/kg BW for RAL immediately after ORX (prophylactically) [OST-P (*n* = 15), RAL-P (*n* = 15), and OST + RAL-P (*n* = 15)] for up to 18 weeks. The dosages of the substances were chosen based on our previous studies [15, 22].

The rats were fed a soy-free rodent diet (ssniff Spezial Diät GmbH, Soest, Germany) throughout the experiment. The rats were housed in cages (Type Makrolon® IV, Techniplast Deutschland GmbH, Hohenpreißenberg, Deutschland), three rats per cage, five cages per treatment group. OST and RAL were supplied with the diet. OST (MK-2866) was obtained from Shanghai Biochempartner Co., Ltd. (Shanghai, China), and RAL was obtained from Evista, Eli Lilly and Company (Indianapolis, USA). All rats had free access to food and demineralized water ad libitum. Weekly records of food intake and BW were maintained. The average daily food intake of a rat was calculated by dividing the food consumed by the number of rats in a cage and it served for the the calculation of the drug intake (OST, RAL, OST + RAL) [23].

Eighteen weeks after ORX, the rats were euthanized after blood collection using a cardiac puncture under deep isoflurane anesthesia. As a marker of muscle damage, creatine kinase (CK) was measured in serum samples [24]. The prostate (both lobes) and three muscles were extracted and weighed: the soleus muscle (SM), the gastrocnemius muscle (GM), and the levator ani muscle (LAM). The SM, GM, and longissimus muscle (LM) were frozen in liquid nitrogen and stored at –80 °C until the muscle fibers and capillaries were analyzed. Either the left or right muscles were used randomly in histological analyses. Contralateral GM was used for the gene expression analyses. The lumbar vertebral body (L4) was scanned using in vivo peripheral quantitative computed tomography (pQCT) at the end of the study to confirm the osteoporotic phenotype and reveal the effect of treatments on bone.

### Histological analyses

Serial cross-sections of 12-µm thickness were cut from the middle part of each muscle with the aid of a cryotome (CM 1900; Leica Microsystems, Wetzler, Germany) at − 20 °C. Specimens were air dried and stored at − 20 °C until staining. All chemicals were obtained from Merck KGaA (Darmstadt, Germany) unless otherwise indicated.

A periodic acid–Schiff (PAS) reaction was applied for staining of muscle capillaries [25]. Briefly, sections were fixed in ethanol/chloroform/glacial acid soluton (16:3:1), incubated in 0.3% α-amylase from porcine pancreas, (Sigma-Aldrich Laborchemikalien GmbH, Seelze, Germany), stained in Schiff’s reagent solution (Roth, Karlsruhe, Germany), and treated with a 10% potassium sulfite solution. Application of Schiff’s reagent solution was performed under visual control to avoid overstaining (2–25 min).

For the analysis of muscle fibers, sections were fixed in a solution of 1% paraformaldehyde solution (pH 6.6), 1% CaCl_2_ and 6% sucrose and then stained by incubation in a reduced nicotinamide adenine dinucleotide diaphorase solution (pH 7.4), followed by acidic incubation (pH 4.2) and incubation in adenosine-5´-triphosphate solution (pH 9.4) [26].

Muscle sections were analyzed using a microscope with a tenfold magnification (Eclipse E 600 microscope; Nikon, Tokyo, Japan), a digital camera (DS-Fi2 Digital Camera; Nikon Instruments Europe, Amsterdam, Netherlands), and digital analysis software (NIS-Elements AR 4.0 imaging software; Nikon Instruments Europe). Three randomly chosen fields of 1 mm^2^ in the ATPase-stained sections were used for the evaluation of fibers. In these fields, 90 slow-twitch oxidative (STO) and fast-twitch oxidative (FTO; fiber types I and IIa, respectively) and 90 fast-twitch glycolytic (FTG; fiber type IIb) fibers were skirted [27]. In the SM, only STO fibers were measured, as the muscle mainly consists of these fibers. MG is relative heterogenic in the distribution pattern of fiber types [28, 29]. Therefore, the fiber distribution was determined solely in ML, as in this muscle, the muscle fibers show a homogenous distribution pattern [30]. The percentage of STO + FTO and FTG fibers was calculated within a 1 mm^2^ field. The capillary density (ratio of capillaries to fibers) was determined in two randomly chosen fields of 0.25 mm^2^ in the amylase-PAS-stained sections [31].

### Gene expression analysis

GM samples (100 mg; *n* = 5/group) were homogenized in 750 µl TRIzol (Thermo Fischer Scientific, WA, USA) using 4 mm tungsten carbide beads (Cat. No. 69997 Qiagen, Germany) with the aid of the Tissuelyzer LT system (Qiagen, Germany). Thereafter, the samples were incubated for 5 min at room temperature and further RNA extraction was processed according to the manufacturer’s protocol (Trizol, Thermo Fischer Scientific) using chloroform and isopropanol treatments and ethanol washings. Finally, the RNA pellet was dissolved in 20 µL H_2_O, measured by DeNovix DS-11 FX + System (DeNovix, NC, USA) and stored at − 80 °C for further analysis.

Reverse transcription was performed with 1000 ng of total RNA using an iScript cDNA synthesis kit (Biorad, CA, USA). Quantitative real-time Polymerase chain reaction (PCR) was performed on the CFX96 Real-time PCR Detection System (Biorad, CA, USA) using a SYBR Green (Biorad, CA, USA) detection marker. Relative expressions of androgen receptor (Ar,), estrogen receptor alpha (Er alpha), myostatin, insulin-like growth factor 1 (Igf-1), and vascular endothelial growth factor B (Vegf-B, [32]) were measured in triplicate and effects were calculated using the 2^−ΔΔCT^ method [33]. Ready-to-use primer were obtained from Qiagen (QuantiTect Primer Assays, Qiagen, Hilden, Germany). Beta-2-microglobulin expression was used as a reference gene. We failed to measure the mRNA expression of Er beta, confirming its low and nearly undetectable expression in the muscles of rodents [34].

### pQCT analysis

L4 was scanned at the end of the trial in isoflurane-anesthetized rats. The overall bone mineral density (BMD, mg/cm^3^), overall bone area (mm^2^), stress–strain index (SSI, mm^3^), and cross-sectional abdomen area (CSA, mm^2^) were calculated. The XCT-6.20C software was used (Stratec Medizintechnik GmbH, Pforzheim, Germany) for data analysis [35].

### Serum analyses

To evaluate the activity of alkaline phosphatase (ALP) and CK, concentration of calcium (Ca), magnesium (Mg), and phosphorus (P) in serum, an automated chemistry analyzer Architect c16000 (Abbott, Wiesbaden, Germany) and commercially available kits (Abbott) were used at the Department of Clinical Chemistry, University of Goettingen, according to the manufacturer’s instructions (Abbott). The following methods were applied: ALP: paranitrolphenyl phosphate method (7D55-30, Abbott) (serum total ALP was determined according to the strong correlation to bone-specific ALP [36]); Ca and Mg: quantification by arsenazi III dye (7D61-20 and 7D70-30, Abbott); P: ammonium molybdate method (7D71-30, Abbott); CK: reactivator method with *N*-acetyl-*L*-cysteine (7D63-30, Abbott) [15].

### Statistical analysis

Statistical analyses were performed with GraphPad Prism ver. 8.2.1 (GraphPad Software, San Diego, CA, USA). A one-way analysis of variance (ANOVA) was used. The differences between the groups were analyzed by applying Tukey’s post-hoc test (*p* < 0.05). Data are presented as means and standard deviations.

## Results

### Experiment I

#### Food and OST intake

The mean food intake did not differ significantly between the treatment groups (Table 1). The mean OST intake was 0.36 ± 0.05 mg/kg BW in the OST-P group and 0.30 ± 0.02 mg/kg BW in the OST-T group [37].

**Table 1:** Statistically non-significant results of Experiment I: Ostarine (OST)

Groups	Non-ORX		ORX		OST-P		OST-T		ANOVA *p* value
Sample size	12		8		7		9		
Parameters	Mean	SD	Mean	SD	Mean	SD	Mean	SD	
**Mean food intake (g/day/rat)**	28.1	8.6	26.9	9.0	27.5	8.8	27.3	8.0	0.977
**Weights**									
Body weight (beginning of trial) [g]	717.5	70.6	713.3	75.3	715.8	71.2	687.9	81.7	0.689
Body weight (end of trial) [g]	727.0	78.2	642	69.5	667.0	104.8	628.7	73.2	0.060
GM weight [g]	3.18	0.46	2.43	1.08	2.87	0.59	3.13	0.41	0.093
GM weight/BW [mg/g]	4.40	0.59	3.85	1.63	4.31	0.58	4.99	0.51	0.141
SM weight [g]	0.27	0.10	0.27	0.05	0.26	0.06	0.30	0.04	0.781
SM weight/BW [mg/g]	0.37	0.14	0.43	0.06	0.40	0.09	0.47	0.04	0.200
**Muscle fibers**									
GM									
STO + FTO									
Area (µm2)	2425	509	2082	478	2640	725	2539	622	0.259
Diameter (µm)	54.6	5.6	50.5	6.3	56.6	7.6	55.9	6.3	0.249
FTG									
Area (µm2)	4432	804	3849	607	4600	1413	4382	739	0.416
Diameter (µm)	74.2	6.6	69.2	5.6	75.1	11.0	73.9	6.1	0.412
LM									
STO + FTO									
Area (µm2)	3107	755	3373	1446	2844	317	3248	640	0.634
Diameter (µm)	61.4	7.2	63.2	12.6	59.2	3.3	62.9	5.8	0.718
FTG									
Area (µm2)	7609	2111	6534	1205	6274	1022	7232	1626	0.249
Diameter (µm)	96.9	12.4	90.1	8.3	88.6	7.2	94.9	10.3	0.244
Percentage of fibers (%)									
STO + FTO	40.6	4.5	41.1	4.8	38.6	5.5	41.7	5.7	0.630
FTG	59.4	4.5	58.9	4.8	61.4	5.5	58.3	5.7	0.630
**Capillary ratio**									
GM	2.0	0.3	1.8	0.2	1.8	0.3	1.8	0.3	0.575
LM	2.0	0.1	1.8	0.2	1.8	0.1	2.0	0.4	0.125
SM	1.6	0.2	1.7	0.3	1.7	0.2	1.6	0.1	0.786
**Expression of genes**									
Vegf-B	1.0	0.4	1.3	0.8	0.8	0.6	0.7	0.9	0.156
**Computer tomography of L4**									
CSA area (mm^2^)	3090	336	2930	360	2754	466	2970	276	0.107
**Serum analysis (U/l)**									
ALP	182.2	53.8	133.7	38	181.6	70.2	194.2	43.3	0.116
Ca	2.2	0.2	2.1	0.2	2.2	0.1	2	0.1	0.241
Mg	0.7	0.1	0.7	0.1	0.7	0.1	0.7	0.1	0.324
CK	6311	1965	5120	1785	5245	1545	4620	1244	0.259

*SD* standard deviation

#### Body, prostate, and muscle weights

The BW of the rats did not change significantly between the treatment groups. Neither ORX nor any of the OST treatments had a significant influence on BW (Table 1) [37].

The prostate weight of the Non-ORX group was significantly higher compared to all other groups. In the OST-P group, prostate weight was higher than in the ORX group (Table 2) [37].

**Table 2:** Statistically significant results of Experiment I: Ostarine (OST)

Groups	Non-ORX			ORX			OST-P			OST-T		ANOVA *p* value
Sample size	12			8			7			9		
Parameters	Mean		SD	Mean		SD	Mean		SD	Mean	SD	
**Weights**												
Prostate weight [g]	1.22^abc^		0.30	0.18^b^		0.07	0.62		0.13	0.39	0.08	< 0.001
LAM weight [g]	0.64^a^		0.25	0.31^bc^		0.08	0.54		0.12	0.57	0.08	0.001
LAM weight/BW [mg/g]	0.90^a^		0.38	0.48^c^		0.12	0.83		0.26	0.92	0.14	0.007
**Muscle fibers**												
SM												
STO												
Area (µm2)	3852		647	4124		615	3382^c^		925	4397	549	0.042
Diameter (µm)	69.2		6.1	71.5		5.3	64.3^c^		8.0	73.9	4.5	0.025
**Expression of genes**												
Ar	1.0		0.1	1.2^b^		0.6	0.8		0.4	1.1	0.3	0.017
Er alpha	1.0^bc^		0.3	0.8		0.4	0.7		0.3	0.7	0.2	0.009
Igf-1	1.0		0.3	0.8^bc^		0.3	1.4		0.8	1.3	0.4	0.001
Myostatin	1.0		0.5	1.2		0.9	1.5^c^		0.8	0.7	0.4	0.032
**Computer tomography of L4**												
Bone density (mg/cm^3^)	475.3^ab^		32.4	430.2^bc^		38.2	526.8^c^		46.1	472.6	50.8	< 0.001
Bone Area (mm^2^)	55.8^b^		8.8	51.6		6.4	48.4		6.4	50.1	5.2	0.027
Stress–Strain-Index	27.0^a^		7.5	21.0		5.2	24.7		3.5	21.9	3.8	0.011
**Serum analysis (U/l)**												
*p*	1.8		0.3	1.6^b^		0.2	2.0		0.3	1.8	0.2	0.003

*SD* standard deviation

Tukey test:

^a^*p* < 0.05 versus ORX

^b^*p* < 0.05 versus OST-P

^c^*p* < 0.05 versus OST-T

Regarding muscle weight, only the LAM weight was significantly lower in the ORX group compared to the Non-ORX, the OST-T, and the OST-P groups [37]. The relative weight of the LAM showed a similar pattern. In the GM and SM groups, neither ORX nor any of the OST treatments significantly changed their absolute or relative weights (Table 1).

#### Muscle structure analysis

In the OST-T group, the area and corresponding diameter of the STO fibers were significantly larger than the OST-P group in the SM (Table 2). In addition, there were no significant alterations of STO/FTO and FTG fibers in the GM and LM across all treatment groups or significant changes in the distribution of muscle fibers in the LM. No significant differences regarding the capillary ratio were observed across the groups in any muscle studied (Table 1). The ratio averaged from 1.8 ± 0.28 in the GM, 1.9 ± 0.22 in the LM, to 1.7 ± 0.20 in the SM (capillaries/muscle fiber).

#### Gene expression

Ar expression was higher in the ORX group compared with the OST-P group (Table 2). Er alpha expression was lower in both OST treatment groups than in the Non-ORX group. Igf-1 was expressed significantly higher in the OST-T and OST-P groups than in the ORX group, and myostatin expression was higher in the OST-P group than in the OST-T group. With regard to Vegf-B, no significant differences were observed (Table 1).

#### pQCT analysis of L4

The highest BMD in the OST-P group and the lowest BMD in the ORX group was revealed among the treatment groups (Table 2). In the Non-ORX group, the bone area was significantly larger than in the OST-P group, and the SSI was higher than in the ORX group. CSA area did not differ between the groups (Table 1).

#### Serum analysis

The OST-P group showed significantly higher P levels than the ORX group. For ALP, Ca, Mg, and CK, no significant differences were observed between the groups (Table 1).

### Experiment II

#### Food, OST, and RAL intake

The mean food intake was lowest in the RAL-P group. The rats in the OST + RAL-P group consumed less than those in the Non-ORX and OST-P groups (Table 3). The mean OST intake was 0.37 ± 0.05 mg/kg BW in the OST-P group and 6.6 ± 1.2 mg/kg BW in the RAL-P group. In the OST + RAL-P group, the average OST intake was 0.40 ± 0.08 mg/kg BW and the average RAL intake was 7.9 ± 1.6 mg/kg BW.

**Table 3:** Statistically significant results of Experiment II: Ostarine (OST), Raloxifen (RAL). Combination (OST + RAL)

Groups	Non-ORX			ORX			OST-P			RAL-P			OST + RAL-P		ANOVA *p* value
Sample size	10			9			9			9			8		
Parameters	Mean		SD	Mean		SD	Mean		SD	Mean		SD	Mean	SD	
**Mean food intake (g/day/rat)**	28.8^cd^		2.7	27.1^c^		4.6	28.6^cd^		4.1	22.5^d^		4.4	25.7	5.8	< 0.001
**Weights**															
Body weight (end of trial) [g]	695.0^acd^		34.3	636.1^cd^		42.3	668.4^cd^		50.3	564.9		47.4	557.0	29.7	< 0.001
Prostate weight [g]	1.27^abcd^		0.38	0.24^b^		0.06	0.63^c^		0.22	0.22^d^		0.10	0.46	0.11	< 0.001
LAM weight [g]	1.91^ac^		0.33	0.91^bd^		0.11	2.00^c^		0.22	0.81^d^		0.12	1.86	0.13	< 0.001
LAM weight/BW [mg/g]	2.75^acd^		0.48	1.43^bd^		0.21	3.02^c^		0.41	1.43^d^		0.18	3.35	0.27	< 0.001
GM weight [g]	3.40^d^		0.39	3.14		0.31	3.18		0.33	3.06		0.41	2.82	0.60	0.016
**Muscle fibers**															
GM															
STO + FTO															
Area (µm2)	2617^abcd^		609	2151		489	2011		514	2153		534	1942	333	< 0.001
Diameter (µm)	57.0^abcd^		6.6	51.6		5.9	49.8		6.1	51.7		6.2	49.2	4.2	< 0.001
FTG															
Area (µm2)	4688		1160	4832^cd^		822	4228		800	4082		863	4082	852	0.008
Diameter (µm)	76.3		9.4	77.8^cd^		6.6	72.7		6.7	71.3		7.5	71.4	7.3	0.007
SM															
STO															
Area (µm2)	4054		559	3976		655	4179		837	4309^d^		587	3647	943	0.025
Diameter (µm)	71.3		4.9	70.5		6.0	72.1		7.5	73.5^d^		5.1	67.3	8.2	0.017
Percentage of fibers in LM (%)															
STO + FTO	47.1		7.1	48.7		7.2	51.9^c^		5.4	41.8		5.0	43.5	7.8	0.018
FTG	53.0		7.1	51.3		7.2	48.1^c^		5.4	58.2		5.0	56.5	7.8	0.018
**Capillary ratio**															
GM	2.2^b^		0.5	2.0		0.2	1.8		0.3	1.9		0.4	2.1	0.4	0.029
SM	2.4		0.5	2.3		0.3	2.6^d^		0.3	2.7^d^		0.3	2.2	0.4	0.001
**Expression of genes**															
Ar	1.0^bcd^		0.1	0.8		0.3	0.6		0.3	0.6		0.1	0.7	0.3	< 0.001
Er alpha	1.0^abd^		0.3	0.7		0.2	0.5^c^		0.2	0.8		0.5	0.7	0.2	< 0.001
Vegf-B	1.0^abc^		0.1	0.7^d^		0.1	0.6^d^		0.3	0.8^d^		0.3	1.8	0.6	< 0.001
Igf-1	1.0^abc^		0.3	0.5		0.1	0.5		0.4	0.6		0.3	0.9	0.5	< 0.001
Myostatin	1.1^b^		0.4	2.1^bcd^		1.7	0.9		0.5	1.4		1.0	1.6	1.6	0.057
**Computer tomography of vertebrae**															
Bone density (mg/cm^3^)	472.7^cd^		53.7	461.4^cd^		60.8	494.4^d^		34.3	523.8		43.2	554.5	49.9	< 0.001
Bone area (mm^2^)	52.7		4.1	48.9^cd^		4.4	51.5^d^		3.1	53.9		4.9	56.0	4.5	< 0.001
Stress–Strain-Index	23.0^d^		5.5	20.3^cd^		4.6	23.2^cd^		2.6	26.3^d^		6.2	32.6	5.5	< 0.001
CSA area (mm^2^)	3167^cd^		189	3045^cd^		284	3030^cd^		290	2563		88	2410	109	< 0.001
**Serum analysis (U/l)**															
ALP	134.0^d^		34.1	137.6		28.5	165.4		45.0	157.9		53.1	199.8	52.0	0.002
Ca	2.2		0.2	2.0^d^		0.2	2.1		0.2	2.1		0.1	2.2	0.2	0.041
P	2.0^abc^		0.2	1.6^d^		0.2	1.8^d^		0.2	1.7^d^		0.2	2.0	0.2	< 0.001

*SD* standard deviation

Tukey test:

^a^*p* < 0.05 versus ORX

^b^*p* < 0.05 versus OST-P

^c^*p* < 0.05 versus RAL-P

^d^*p* < 0.05 versus OST + RAL-P

#### Body, prostate, and muscle weights

At the beginning of the trial, the BW did not differ significantly across treatment groups, whereas at the end of the trial, the RAL-P and OST + RAL-P treatment groups showed a significantly lower BW compared with the Non-ORX, the ORX, and the OST-P groups. Furthermore, at the end of the trial, the BW of the ORX group was lower than that of the Non-ORX group (Table 3).

The prostate weight of the Non-ORX rats was significantly higher compared to all other treatment groups. The OST-P group showed a higher prostate weight than the ORX and RAL-P groups. The prostate weight of the RAL-P group remained at the level of ORX rats, i.e., it was significantly lower compared with the OST-P, the OST + RAL-P, and the Non-ORX groups (Table 3).

Regarding muscle weights, the absolute and relative LAM weights of the ORX and RAL-P groups were significantly lower than those of the Non-ORX, OST-P, and OST + RAL-P groups. Additionally, the relative LAM weight was higher in the OST + RAL-P group than in the Non-ORX group. GM weight was higher in the Non-ORX group than in the OST + RAL-P groups (Table 3). The relative and absolute weights of SM did not differ between the groups (Table 4).

**Table 4:** Statistically non-significant results of Experiment II: Ostarine (OST), Raloxifen (RAL), Combination (OST + RAL)

Groups	Non-ORX		ORX		OST-P		RAL-P		OST + RAL-P		ANOVA *p* value
Sample size	`10		9		9		9		8		
Parameters	Mean	SD	Mean	SD	Mean	SD	Mean	SD	Mean	SD	
**Weights**											
Body weight (beginning of trial) [g]	631.5	22.7	624.9	35.0	637.9	23.4	640.4	29.3	631.5	20.2	0.539
GM weight/BW [mg/g]	4.90	0.60	4.57	1.42	4.44	1.07	5.03	1.55	5.05	0.99	0.585
SM weight [g]	0.29	0.03	0.27	0.03	0.29	0.04	0.27	0.05	0.24	0.07	0.055
SM weight/BW [mg/g]	0.42	0.05	0.39	0.13	0.40	0.11	0.48	0.09	0.44	0.12	0.216
**Muscle fibers**											
LM											
STO + FTO											
Area (µm^2^)	1789	371	1627	366	1645	366	1778	450	1601	407	0.243
Diameter (µm)	47.3	4.9	45.0	4.9	45.3	5.0	46.9	6.0	44.7	5.2	0.236
FTG											
Area (µm^2^)	5997	1584	5993	1427	5906	1416	6235	1411	5656	758	0.671
Diameter (µm)	86.4	11.5	86.5	10.1	85.8	9.9	88.2	10.2	84.4	5.7	0.735
**Capillary ratio**											
LM	1.7	0.3	1.9	0.4	1.7	0.4	1.8	0.4	2.0	0.4	0.085
**Serum analysis (U/l)**											
Mg	0.8	0.1	0.7	0.1	0.7	0.1	0.7	0.1	0.7	0.1	0.157
CK	5715	1844	5568	2657	5920	3310	6530	2722	7494	4675	0.509

*SD* dtandard deviation

#### Muscle analysis

In the GM, both the area and the corresponding diameter of STO/FTO fibers in the Non-ORX group were significantly larger compared to all other treatment groups (Table 3). The FTG fibers of the GM showed a significantly larger area and diameter in the ORX group than in the RAL-P and OST + RAL-P groups. The LM showed no significant differences in muscle fiber size (Table 4). In the SM, both the area and the corresponding diameter of the fibers in the RAL-P group were significantly larger than in the OST + RAL-P group. With regard to the distribution of muscle fibers in LM, there were significantly more STO/FTO fibers and significantly fewer FTG fibers in the OST-P group than in the RAL-P group (Table 3).

In the GM, the capillary ratio was significantly higher in the Non-ORX group compared to the OST-P group. In the SM group, the OST-P and RAL-P groups showed significantly higher capillary ratios than the OST + RAL-P group (Table 3). No significant differences were observed in LM (Table 4).

#### Gene expression

In the GM, Ar expression was significantly higher in the Non-ORX group than in the OST-P, RAL-P, and OST + RAL-P groups. Er alpha was expressed significantly higher in the Non-ORX group than in the ORX, OST-P, and OST + RAL-P groups. In the RAL-P group, Er alpha expression was significantly higher than in the OST-P group. Vegf-B showed a higher expression in the Non-ORX group than in the OST-P group. Furthermore, Vegf-B expression was higher in the OST + RAL-P group than in the ORX, OST-P, and RAL-P groups. Igf-1 was expressed higher in the Non-ORX group than in the ORX, OST-P, and RAL-P groups. Myostatin expression was higher in the ORX group than in all other groups (Table 3).

#### pQCT analysis of L4

The BMD was significantly higher in the OST + RAL-P group compared with the Non-ORX, ORX, and OST-P groups. The RAL-P group showed a significantly higher BMD than the Non-ORX and ORX groups. The area was significantly larger in the OST + RAL-P group than in the ORX and OST-P groups, and significantly larger in the RAL-P group than in the Non-ORX group. The SSI had a significantly higher value in the OST + RAL-P group than in all other treatment groups. Furthermore, the SSI was significantly higher in the RAL-P group than in the ORX and OST-P groups. The CSA was smaller in the OST + RAL-P and RAL-P groups than in the non-ORX, ORX, and OST-P groups (Table 3).

#### Serum analysis

The ALP activity in the OST + RAL-P group was significantly higher compared to the Non-ORX group. The OST + RAL-P group showed significantly higher Ca levels than the ORX group. P levels were significantly higher in the Non-ORX and the OST + RAL-P groups than in the ORX, OST-P, and RAL-P groups (Table 3). The serum CK and Mg levels did not differ across the groups (Table 4).

## Discussion

In the present study, the effects of SARM ostarine and SERM raloxifen on muscle tissue were evaluated in an orchiectomized rat model, reflecting a hormone-dependent decline in the musculoskeletal system. To our knowledge, we report for the first time the influence of the combined treatment of these selective receptor modulators on musculoskeletal tissue.

With regard to muscle weight, treatment with OST showed major effects on LA. In both experiments, LAM weight was reduced after ORX, while OST therapy reversed this effect, as shown in previous studies [38, 39]. This indicates an anabolic effect of OST on LA, similar to that observed for testosterone. Testosterone was demonstrated to exert strong anabolic effects on LA, whereas no changes were found in the weights of the soleus, extensor digitorum longus, and diaphragm muscles [40]. In contrast, the weight of GM was enhanced after treatment with OST at the same dose in an ovariectomized rat model [37]. It is possible that the chosen dose of OST was sufficient to induce changes in the female rat model but did not exert an effect on muscle weight in male rats. Thus, gender differences should be considered when applying SARM treatments to induce skeletal muscle response. It is known that normal endogenous serum total testosterone concentrations in women are lower than those of healthy men [41].

Treatment with RAL did not restore muscle weight loss after ORX in the LAM group. In the GM, a slight decline was detected in the RAL group, and the combination of OST and RAL resulted in a significant decrease in GM weight. This was probably due to the differences in the BW of the rats in these groups, since this effect was not found in related data. A strong correlation between BW and muscle weight was previously shown [42]. Favorable effects of SERMs, including RAL, on skeletal muscle function and structure have been reported, whereas no changes in muscle weight have been observed in mice with muscular dystrophy [10, 11]. While the underlying mechanism remains unclear, the inhibition of fibrosis, protection against contraction, oxidative stress, mitochondria-mediated cell death, or calcium regulation are discussed [10, 11].

Reduction in muscle weight reflected changes observed in BW after both mono and combined treatments with RAL. It has been shown that RAL decreased BW in female ovariectomized rats, possibly by regulating Wnt and inhibition of adipogenesis [43]. In line with this would be the reduced CSA that was observed in the RAL and OST + RAL groups, while the RAL group showed decreased food intake in the present experiment. Considering the fact that another SARM enhanced intramuscular fat content [15], the reduction in muscle weight under combined treatment, along with a decrease in CSA, which indirectly indicates the size of the abdominal fat depot [44], could be considered a positive effect of OST + RAL treatment on orchiectomized rat physiology.

In both experiments, treatment with OST had an androgenic effect on the prostate, which was stronger after prolonged prophylactic administration (P) than after short therapeutic administration (T) in Experiment I. In contrast, RAL treatment did not affect the prostate weight in ORX rats but diminished the androgenic effect of OST on prostate weight in the combination therapy. Both effects—enhancement of prostate weight in castrated rats by OST and the described antiprostatic-like effects of RAL—have been reported previously [45, 46]. However, to our knowledge, the reduction of the androgenic effect of OST in combination with RAL has not yet been reported.

While prophylactic administration of OST showed only minimal effects on muscle fiber size in both experiments, therapeutic administration of OST increased muscle fiber size in the SM group compared to the OST-P group (Experiment I). This might indicate a time dependency and a difference between the P and T administration approaches. According to this, Jones, Hwang [47] showed an increase in the muscle atrophy F-box (*MAFbx*, FBXO32) in the first two weeks after the castration of rats, which might interfere with early (prophylactic) OST treatment. Furthermore, Vyskocil and Gutmann [40] observed a stronger effect of shortly administered testosterone on muscle weight compared to a longer period of testosterone administration. In Experiment II, RAL administration resulted in a smaller muscle fiber size of the GM. This effect was not alleviated by combination therapy. Wu, Shah [11] observed a normalizing effect of RAL on the muscle fiber size of mice with muscle dystrophy. However, the long-term administration of RAL did not result in increased muscle fiber size. In our experiment, the changes in muscle fiber size could be explained by the decrease in BW under RAL treatment. As previously reported, both muscle weight and muscle fiber size correlated with body weight in a rat model [42]. In the GM, ORX resulted in a smaller muscle fiber size compared with the Non-ORX group, confirming the effect of ORX [38]. However, neither OST nor RAL administration could reverse this effect. OST-P treatment showed an enhanced percentage of oxidative fibers in the Experiment II, which reached a significant level in comparison to the RAL-P treatment. In a rabbit model of high fat diet-induced metabolic syndrome, testosterone treatment was able to prevent a shift from oxidative fibers to glycolytic fibers [48]. This may indicate, that OST acts similar as testosterone on muscle composition. However, further analyses are required to confirm this observation.

The capillary ratio was only marginally affected by OST or RAL administration in both experiments. In contrast, a previous study that examined OST therapeutic administration in ovariectomized rats showed an increase of the capillary ratio in skeletal muscles [15] that could be explained by enhanced sensitivity of female muscle to OST compared to male muscle. Similar to our results, RAL, and estrogen did not change the capillary ratio in the skeletal muscles of the ovariectomized rats [49, 50].

Gene expression analysis showed increased expression of Vegf-B under combination therapy. Vegf-B controls vascularization in muscle [51]; whether its mRNA increase would result in an improvement of capillarization during prolonged treatment remains unexplored.

Igf-1 gene expression was enhanced after both OST treatments (P and T) in Experiment I, which could indicate the positive anabolic action of SARM on muscle. A decrease of Igf-1 expression upon castration and its increase after OST and dihydrotestosterone administration have been previously reported in ORX mice [39]. However, in Experiment II, OST administration did not restore Igf-1 expression in the ORX rats. Similarly, RAL did not change the decreased level of Igf-1 in muscle. Nevertheless, under combination therapy, Igf-1 expression was restored to the level of Non-ORX healthy rats, which could be a positive sign for this treatment.

Myostatin, a negative regulator of muscle growth [52, 53] was expressed at a higher level in ORX rats and diminished after both OST treatments (T and P) as well as after combination treatment. This confirmed a muscle anabolic effect of OST [13, 14].

Ar gene expression in GM after ORX was not significantly altered, corresponding to earlier results [38], whereas OST and RAL decreased Ar expression. In contrast, administration of testosterone caused an increase in Ar expression in skeletal muscles [38]. This could be explained by the partial aromatization of testosterone into estrogen, which was reported to induce upregulation of Ar expression [54].

The diminished Er alpha expression after ORX was not changed by OST, whereas RAL administration restored it to the level of Non-ORX rats. Increasing expression of Er alpha has been observed after RAL administration in human skeletal muscle [55] that indicates its influence on the muscle through Er.

Both experiments showed favorable effects of OST and RAL on bone. The BMD was higher after prophylactic OST and RAL administration and the combination treatment, which is in line with results that showed the beneficial effects of sole OST or RAL administration on the BMD of rats [56–58]. The osteoanabolic and protective effects of OST have been previously reported [8, 13, 57]. In Experiment II, RAL had a positive impact on SSI. The favorable effect of SERMs on bone is explained by their inhibition of osteoclast activity [59]. The strongest effect on bone was observed in the OST + RAL-P group compared to all other groups. Beneficial actions of combination therapies have been reported for teriparatide and RAL in clinical conditions in postmenopausal women [60].

The serum analysis revealed significantly elevated ALP levels in the OST + RAL-P group compared to the Non-ORX group, whereas neither OST nor RAL administration changed its level. Although the role of ALP is not yet entirely understood, it is thought to be crucial in bone metabolism [36]. Our group recently reported a dose-dependent positive impact of OST on serum ALP levels [56, 58], and Furuya, Yamamoto [21] showed that SARM S-101479 increased ALP expression. Regarding the influence of RAL on ALP, increased expression was also observed after its administration [61, 62]. Furthermore, in the present study, only the combination of OST and RAL could restore serum P and Ca levels to the level of the Non-ORX group, while it decreased in all other treatment groups. Hypophosphatemia and -calcemia are associated with strong disorders, and electrolyte balance needs to be maintained [63]. Although an increase in serum P levels has been reported after sole OST [37, 56] or RAL [62] administration in ovariectomized rats, this study showed a favorable effect of combination therapy. The results might once again indicate the advantage of combination therapy compared with the sole administration of OST or RAL. CK was not affected by either treatment, indicating the lack of severe detrimental changes in muscle tissue [64].

The study has several limitations as follows: 1) The study focused on muscle metabolism, structure, and mRNA expression, while it lacks the functional analysis of muscle force. This analysis should be addressed in the following projects to evaluate the direct physical effect of the therapies. 2) Besides myostatin and Igf-1, further myogenic markers such as paired box 7, myogenin or mygenic factor 5 should be included in the future studies to better understand the anabolic effects of the treatments [48]. 3) The analysis of fiber type composition could be extended by the analysis of mRNA expression of myosin heavy chain isoforms [48]. 4) The study analyzes the results of two independent experiments and some data (*e.g*. gene expression) showed discrepancies due to the unknown circumstances that probably occurred during experiment, sample collection or data analyses.

Summarizing, sole OST-P treatment exerted favorable effects on muscles, particularly on the LA; however, it was associated with an androgenic impact on the prostate in terms of weight gain. OST-T administration partially increased favorable effects and diminished androgenic effects compared with P administration. Sole RAL-P treatment did not show relevant anabolic effects on muscles but improved body constitution by reducing CSA, food intake, and BW. The most favorable effects were observed after the combined treatment of OST and RAL. While the anabolic impact on muscle structure and metabolism was maintained, the androgenic potential in the prostate was reduced. Furthermore, the food intake was normalized, although the CSA remained decreased. Both OST and RAL had a favorable effect, and their combination showed a synergetic and positive impact on bone tissue. Thus, OST + RAL treatment is promising for the treatment of musculoskeletal tissue under androgen deficient conditions and shows fewer side effects than the respective monotherapies. Nevertheless, it is noteworthy that these results refer to orchiectomized rats and still many issues have to be analyzed before a possible application in humans.

## References

[1] Giannoulis MG, Martin FC, Nair KS, Umpleby AM, Sonksen P (2012). Hormone replacement therapy and physical function in healthy older men. Time to talk hormones?. Endocr Rev.

[2] Huang L-T, Wang J-H (2021). The therapeutic intervention of sex steroid hormones for sarcopenia. Front Med.

[3] Horstman AM, Dillon EL, Urban RJ, Sheffield-Moore M (2012). The role of androgens and estrogens on healthy aging and longevity. J Gerontol A.

[4] Barbonetti A, D'Andrea S, Francavilla S (2020). Testosterone replacement therapy. Andrology.

[5] Palacios S, Mejias A (2015). An update on drugs for the treatment of menopausal symptoms. Expert Opin Pharmacother.

[6] Stuenkel CA (2015). Menopausal hormone therapy: current considerations. Endocrinol Metab Clin N Am.

[7] Rossouw JE, Anderson GL, Prentice RL, LaCroix AZ, Kooperberg C, Stefanick ML, Jackson RD, Beresford SA, Howard BV, Johnson KC, Kotchen JM, Ockene J, I. Writing Group for the Women's Health Initiative (2002). Risks and benefits of estrogen plus progestin in healthy postmenopausal women: principal results From the Women's Health Initiative randomized controlled trial. JAMA.

[8] Bhasin S, Calof OM, Storer TW, Lee ML, Mazer NA, Jasuja R, Montori VM, Gao W, Dalton JT (2006). Drug insight: testosterone and selective androgen receptor modulators as anabolic therapies for chronic illness and aging. Nat Clin Pract Endocrinol Metab.

[9] Christiansen AR, Lipshultz LI, Hotaling JM, Pastuszak AW (2020). Selective androgen receptor modulators: the future of androgen therapy?. Transl Androl Urol.

[10] Dorchies OM, Reutenauer-Patte J, Dahmane E, Ismail HM, Petermann O, Patthey- Vuadens O, Comyn SA, Gayi E, Piacenza T, Handa RJ, Decosterd LA, Ruegg UT (2013). The anticancer drug tamoxifen counteracts the pathology in a mouse model of duchenne muscular dystrophy. Am J Pathol.

[11] Wu B, Shah SN, Lu P, Bollinger LE, Blaeser A, Sparks S, Harper AD, Lu QL (2018). Long-term treatment of tamoxifen and raloxifene alleviates dystrophic phenotype and enhances muscle functions of FKRP dystroglycanopathy. Am J Pathol.

[12] Crawford J, Prado CM, Johnston MA, Gralla RJ, Taylor RP, Hancock ML, Dalton JT (2016). Study design and rationale for the phase 3 clinical development program of enobosarm, a selective androgen receptor modulator, for the prevention and treatment of muscle wasting in cancer patients (POWER Trials). Curr Oncol Rep.

[13] Dalton JT, Barnette KG, Bohl CE, Hancock ML, Rodriguez D, Dodson ST, Morton RA, Steiner MS (2011). The selective androgen receptor modulator GTx-024 (enobosarm) improves lean body mass and physical function in healthy elderly men and postmenopausal women: results of a double-blind, placebo-controlled phase II trial. J Cachexia Sarcopenia Muscle.

[14] Dobs AS, Boccia RV, Croot CC, Gabrail NY, Dalton JT, Hancock ML, Johnston MA, Steiner MS (2013). Effects of enobosarm on muscle wasting and physical function in patients with cancer: a double-blind, randomised controlled phase 2 trial. Lancet Oncol.

[15] Roch PJ, Henkies D, Carstens JC, Krischek C, Lehmann W, Komrakova M, Sehmisch S (2020). Ostarine and ligandrol improve muscle tissue in an ovariectomized rat model. Front Endocrinol (Lausanne).

[16] Duschek EJ, Gooren LJ, Netelenbos C (2004). Effects of raloxifene on gonadotrophins, sex hormones, bone turnover and lipids in healthy elderly men. Eur J Endocrinol.

[17] Uebelhart B, Herrmann F, Pavo I, Draper MW, Rizzoli R (2004). Raloxifene treatment is associated with increased serum estradiol and decreased bone remodeling in healthy middle-aged men with low sex hormone levels. J Bone Miner Res.

[18] Jacobsen DE, Samson MM, Emmelot-Vonk MH, Verhaar HJ (2010). Raloxifene and body composition and muscle strength in postmenopausal women: a randomized, double-blind, placebo-controlled trial. Eur J Endocrinol.

[19] Cheung AS, Zajac JD, Grossmann M (2014). Muscle and bone effects of androgen deprivation therapy: current and emerging therapies. Endocr Relat Cancer.

[20] Kamel HK, Maas D, Duthie EH (2002). Role of hormones in the pathogenesis and management of sarcopenia. Drugs Aging.

[21] Furuya K, Yamamoto N, Ohyabu Y, Morikyu T, Ishige H, Albers M, Endo Y (2013). Mechanism of the tissue-specific action of the selective androgen receptor modulator S-101479. Biol Pharm Bull.

[22] Stuermer EK, Sehmisch S, Tezval M, Tezval H, Rack T, Boekhoff J, Wuttke W, Herrmann TR, Seidlova-Wuttke D, Stuermer KM (2009). Effect of testosterone, raloxifene and estrogen replacement on the microstructure and biomechanics of metaphyseal osteoporotic bones in orchiectomized male rats. World J Urol.

[23] Komrakova M, Rechholtz C, Pohlmann N, Lehmann W, Schilling AF, Wigger R, Sehmisch S, Hoffmann DB (2019). Effect of alendronate or 8-prenylnaringenin applied as a single therapy or in combination with vibration on muscle structure and bone healing in ovariectomized rats. Bone Rep.

[24] Brancaccio P, Maffulli N, Limongelli FM (2007). Creatine kinase monitoring in sport medicine. Br Med Bull.

[25] Andersen P (1975). Capillary density in skeletal muscle of man. Acta Physiol Scand.

[26] Horak V (1983). A successive histochemical staining for succinate dehydrogenase and "reversed"-ATPase in a single section for the skeletal muscle fibre typing. Histochemistry.

[27] Peter JB, Barnard RJ, Edgerton VR, Gillespie CA, Stempel KE (1972). Metabolic profiles of three fiber types of skeletal muscle in guinea pigs and rabbits. Biochemistry.

[28] Armstrong RB, Phelps RO (1984). Muscle fiber type composition of the rat hindlimb. Am J Anat.

[29] Staron RS, Kraemer WJ, Hikida RS, Fry AC, Murray JD, Campos GE (1999). Fiber type composition of four hindlimb muscles of adult Fisher 344 rats. Histochem Cell Biol.

[30] Schwartz-Giblin S, Rosello L, Pfaff DW (1983). A histochemical study of lateral longissimus muscle in rat. Exp Neurol.

[31] Saul D, Harlas B, Ahrabi A, Kosinsky RL, Hoffmann DB, Wassmann M, Wigger R, Boker KO, Sehmisch S, Komrakova M (2018). Effect of strontium ranelate on the muscle and vertebrae of ovariectomized rats. Calcif Tissue Int.

[32] Spandidos A, Wang X, Wang H, Seed B (2010). PrimerBank: a resource of human and mouse PCR primer pairs for gene expression detection and quantification. Nucleic Acids Res.

[33] Livak KJ, Schmittgen TD (2001). Analysis of relative gene expression data using real-time quantitative PCR and the 2(-Delta Delta C(T)) Method. Methods.

[34] Couse JF, Lindzey J, Grandien K, Gustafsson JA, Korach KS (1997). Tissue distribution and quantitative analysis of estrogen receptor-alpha (ERalpha) and estrogen receptor-beta (ERbeta) messenger ribonucleic acid in the wild-type and ERalpha-knockout mouse. Endocrinology.

[35] Saul D, Gleitz S, Nguyen HH, Kosinsky RL, Sehmisch S, Hoffmann DB, Wassmann M, Menger B, Komrakova M (2017). Effect of the lipoxygenase-inhibitors baicalein and zileuton on the vertebra in ovariectomized rats. Bone.

[36] Seibel MJ (2005). Biochemical markers of bone turnover: part I: biochemistry and variability. Clin Biochem Rev.

[37] Komrakova M, Nagel J, Hoffmann DB, Lehmann W, Schilling AF, Sehmisch S (2020). Effect of selective androgen receptor modulator enobosarm on bone healing in a rat model for aged male osteoporosis. Calcif Tissue Int.

[38] Antonio J, Wilson JD, George FW (1999). Effects of castration and androgen treatment on androgen-receptor levels in rat skeletal muscles. J Appl Physiol (1985).

[39] Dubois V, Simitsidellis I, Laurent MR, Jardi F, Saunders PT, Vanderschueren D, Claessens F (2015). Enobosarm (GTx-024) modulates adult skeletal muscle mass independently of the androgen receptor in the satellite cell lineage. Endocrinology.

[40] Vyskocil F, Gutmann E (1977). Anabolic effect of testosterone on the levator ani muscle of the rat. Pflugers Arch.

[41] Dillon EL, Durham WJ, Urban RJ, Sheffield-Moore M (2010). Hormone treatment and muscle anabolism during aging: androgens. Clin Nutr.

[42] Komrakova M, Hoffmann DB, Nuehnen V, Stueber H, Wassmann M, Wicke M, Tezval M, Stuermer KM, Sehmisch S (2016). The effect of vibration treatments combined with teriparatide or strontium ranelate on bone healing and muscle in ovariectomized rats. Calcif Tissue Int.

[43] Shen HH, Yang CY, Kung CW, Chen SY, Wu HM, Cheng PY, Lam KK, Lee YM (2019). Raloxifene inhibits adipose tissue inflammation and adipogenesis through Wnt regulation in ovariectomized rats and 3 T3–L1 cells. J Biomed Sci.

[44] Seidlová-Wuttke D, Stürmer KM, Stürmer EK, Sehmisch S, Wuttke W (2006). Contrasting effects of estradiol, testosterone and of a black cohosh extract on density, mechanical properties and expression of several genes in the metaphysis of the tibia and on fat tissue of orchidectomized rats. Maturitas.

[45] Neubauer BL, Best KL, Clemens JA, Gates CA, Goode RL, Jones CD, Laughlin ME, Shaar CJ, Toomey RE, Hoover DM (1993). Endocrine and antiprostatic effects of raloxifene (LY156758) in the male rat. Prostate.

[46] Yin D, Gao W, Kearbey JD, Xu H, Chung K, He Y, Marhefka CA, Veverka KA, Miller DD, Dalton JT (2003). Pharmacodynamics of selective androgen receptor modulators. J Pharmacol Exp Ther.

[47] Jones A, Hwang DJ, Narayanan R, Miller DD, Dalton JT (2010). Effects of a novel selective androgen receptor modulator on dexamethasone-induced and hypogonadism-induced muscle atrophy. Endocrinology.

[48] Sarchielli E, Comeglio P, Filippi S, Cellai I, Guarnieri G, Guasti D, Rapizzi E, Rastrelli G, Bani D, Vannelli G, Vignozzi L, Morelli A, Maggi M (2020). Testosterone improves muscle fiber asset and exercise performance in a metabolic syndrome model. J Endocrinol.

[49] Komrakova M, Werner C, Wicke M, Nguyen BT, Sehmisch S, Tezval M, Stuermer KM, Stuermer EK (2009). Effect of daidzein, 4-methylbenzylidene camphor or estrogen on gastrocnemius muscle of osteoporotic rats undergoing tibia healing period. J Endocrinol.

[50] Schiefer S (2018). Einfluss der Ganzkörpervibration in Kombination mit Östrogen und Raloxifen auf die Skelettmuskulatur der ovarektomierten Ratte (Influence of whole-body vibration in combination with estrogen and raloxifene on the skeletal muscles of the ovariectomized rat). Klinik für Unfallchirurgie, Orthopädie und Plastische Chirurgie.

[51] Wagner PD (2011). The critical role of VEGF in skeletal muscle angiogenesis and blood flow. Biochem Soc Trans.

[52] Jasuja R, LeBrasseur NK (2014). Regenerating skeletal muscle in the face of aging and disease. Am J Phys Med Rehabil.

[53] Roth SM, Walsh S (2004). Myostatin: a therapeutic target for skeletal muscle wasting. Curr Opin Clin Nutr Metab Care.

[54] Shao R, Ljungstrom K, Weijdegard B, Egecioglu E, Fernandez-Rodriguez J, Zhang FP, Thurin-Kjellberg A, Bergh C, Billig H (2007). Estrogen-induced upregulation of AR expression and enhancement of AR nuclear translocation in mouse fallopian tubes in vivo. Am J Physiol Endocrinol Metab.

[55] Dieli-Conwright CM, Spektor TM, Rice JC, Todd Schroeder E (2009). Oestradiol and SERM treatments influence oestrogen receptor coregulator gene expression in human skeletal muscle cells. Acta Physiol (Oxf).

[56] Hoffmann DB, Komrakova M, Pflug S, von Oertzen M, Saul D, Weiser L, Walde TA, Wassmann M, Schilling AF, Lehmann W, Sehmisch S (2019). Evaluation of ostarine as a selective androgen receptor modulator in a rat model of postmenopausal osteoporosis. J Bone Miner Metab.

[57] Kearbey JD, Gao W, Narayanan R, Fisher SJ, Wu D, Miller DD, Dalton JT (2007). Selective Androgen Receptor Modulator (SARM) treatment prevents bone loss and reduces body fat in ovariectomized rats. Pharm Res.

[58] Komrakova M, Furtwangler J, Hoffmann DB, Lehmann W, Schilling AF, Sehmisch S (2020). The selective androgen receptor modulator ostarine improves bone healing in ovariectomized rats. Calcif Tissue Int.

[59] Migliaccio S, Brama M, Spera G (2007). The differential effects of bisphosphonates, SERMS (selective estrogen receptor modulators), and parathyroid hormone on bone remodeling in osteoporosis. Clin Interv Aging.

[60] Deal C, Omizo M, Schwartz EN, Eriksen EF, Cantor P, Wang J, Glass EV, Myers SL, Krege JH (2005). Combination teriparatide and raloxifene therapy for postmenopausal osteoporosis: results from a 6-month double-blind placebo-controlled trial. J Bone Miner Res.

[61] Lin Y, Liu LJ, Murray T, Sodek J, Rao L (2004). Effect of raloxifene and its interaction with human PTH on bone formation. J Endocrinol Invest.

[62] Canpolat S, Tug N, Seyran AD, Kumru S, Yilmaz B (2010). Effects of raloxifene and estradiol on bone turnover parameters in intact and ovariectomized rats. J Physiol Biochem.

[63] Moe SM (2008). Disorders involving calcium, phosphorus, and magnesium. Prim Care.

[64] Komulainen J, Kytola J, Vihko V (1994). Running-induced muscle injury and myocellular enzyme release in rats. J Appl Physiol.

